# PTSD or not PTSD? Comparing the proposed ICD-11 and the DSM-5 PTSD criteria among young survivors of the 2011 Norway attacks and their parents

**DOI:** 10.1017/S0033291716002968

**Published:** 2017-01-12

**Authors:** G. S. Hafstad, S. Thoresen, T. Wentzel-Larsen, A. Maercker, G. Dyb

**Affiliations:** 1 Norwegian Centre for Violence and Traumatic Stress Studies, Pb. 181 Nydalen, 0409 Oslo, Norway; 2 Department of Psychology – Psychopathology and Clinical Intervention, University of Zurich,Binzmühlestrasse 14/17, 8050 Zürich, Switzerland

**Keywords:** DSM-5, ICD-11, post-traumatic stress disorder, prevalence

## Abstract

**Background:**

The conceptualization of post-traumatic stress disorder (PTSD) in the upcoming International Classification of Diseases (ICD)-11 differs in many respects from the diagnostic criteria in the Diagnostic and Statistical Manual for Mental Disorders, fifth edition (DSM-5). The consequences of these differences for individuals and for estimation of prevalence rates are largely unknown. This study investigated the concordance of the two diagnostic systems in two separate samples at two separate waves.

**Method:**

Young survivors of the 2011 Norway attacks (*n* = 325) and their parents (*n* = 451) were interviewed at 4–6 months (wave 1) and 15–18 months (wave 2) after the shooting. PTSD was assessed with the UCLA PTSD Reaction Index for DSM-IV adapted for DSM-5, and a subset was used as diagnostic criteria for ICD-11.

**Results:**

In survivors, PTSD prevalence did not differ significantly at any time point, but in parents, the DSM-5 algorithm produced significantly higher prevalence rates than the ICD-11 criteria. The overlap was fair for survivors, but amongst parents a large proportion of individuals met the criteria for only one of the diagnostic systems. No systematic differences were found between ICD-11 and DSM-5 in predictive validity.

**Conclusions:**

The proposed ICD-11 criteria and the DSM-5 criteria performed equally well when identifying individuals in distress. Nevertheless, the overlap between those meeting the PTSD diagnosis for both ICD-11 and DSM-5 was disturbingly low, with the ICD-11 criteria identifying fewer people than the DSM-5. This represents a major challenge in identifying individuals suffering from PTSD worldwide, possibly resulting in overtreatment or unmet needs for trauma-specific treatment, depending on the area of the world in which patients are being diagnosed.

## Introduction

While the International Classification of Diseases (ICD) is the official clinical diagnostic system in use worldwide, apart from the USA, most research is based on the Diagnostic and Statistical Manual for Mental Disorders (DSM). Both diagnostic systems have recently been revised; the DSM-5 (American Psychiatric Association, 2013) was published in 2013 and the ICD-11 is due in 2018 (Maercker *et al.* 2013*b*). Despite efforts to harmonize the DSM-5 and ICD-11, varying perspectives on post-traumatic stress disorder (PTSD) have led to divergent criteria, and, as a result, the classifications differ in several ways. The DSM-5 adopted a broad approach, aiming to cover a wide range of clinically relevant criteria, while the ICD-11 has moved in the opposite direction. Because scholars have questioned the reliability of the long DSM-5 symptom list and its complex diagnostic algorithm (Galatzer-Levy & Bryant, 2013), the current ICD-11 proposal intends to improve clinical utility of the classification system, taking into account different prerequisites and settings for mental health providers (Maercker & Perkonigg, 2013). The current approach therefore aims to identify the core features of PTSD by excluding symptoms that overlap with other disorders, such as depression (Maercker *et al.* 2013*a*). As such, the ICD-11 proposal suggests retaining the three symptom clusters from the ICD-10, i.e. re-experiencing, avoidance and hyperarousal, but reducing the number of symptoms within each cluster to two. While memories of the traumatic event (TE) may be considered a distinct feature specific to trauma reactions, intrusive recollection of events is also shown to be a common symptom of depression as well as a number of other psychiatric disorders (Brewin *et al.* 2010). For this reason, the ICD-11 work group has not yet agreed on whether to include it as a core symptom. In this paper, we aim to elucidate this issue by comparing two alternative proposed ICD-11 models, only one of which includes intrusive memories.

To date, only a handful of studies have evaluated the differences between the DSM-5 and ICD-11 criteria, generally showing that the DSM-5 criteria provide significantly higher prevalence figures than the ICD-11 criteria. This was true for a multisite study of predominantly male severely injured patients (O'Donnell *et al.* 2014), one clinical sample of treatment-seeking adult survivors of sexual abuse (Hyland *et al.* 2016), two veteran samples (Wisco *et al.* 2016) and finally in a large sample comprising seven different trauma samples (Hansen *et al.* 2015). In contrast, a cross-national epidemiological study reported no significant difference in prevalence between the two systems (Stein *et al.* 2014).

Since the classifications of PTSD differ in several ways, the diagnostic approaches may identify different groups of individuals as having PTSD. For example, someone diagnosed with PTSD in the USA would not necessarily be considered to have PTSD in Europe. A large diagnostic overlap could justify two parallel systems for identifying PTSD, but discrepancies will naturally raise questions about PTSD as a consistent and reliable phenomenon.

Prevalence rates are important, but they provide little information about the clinical utility of the diagnostic criteria. One can argue that diagnostic criteria become relevant when they are able to identify individuals whose mental health or functioning is impaired in one or more areas of life. O'Donnell *et al.* (2014) found that the differences between the diagnoses were few, but that ICD-11 showed a lower co-morbidity with depression and lower sensitivity for detecting disability and reduced life satisfaction than DSM-5. Introducing two divergent classifications of PTSD calls for thorough evaluation of the similarities and differences between the diagnostic outcomes. Therefore, the current study had three overall aims:

(1) To investigate the concordance between the DSM-5 and the proposed ICD-11 PTSD diagnostic algorithms in young survivors of a mass shooting and their parents.
(2) To evaluate the two alternative algorithms for identifying PTSD in ICD-11, one including intrusive memories as a third core symptom of re-experiencing, and one including only flashbacks and nightmares in this category.
(3) To assess the ability of the two diagnostic systems to predict levels of anxiety/depression, functional impairment and life satisfaction.

## Method

### Participants and procedures

The police registered 495 survivors of the terrorist attack which occurred on 22 July 2011, when a heavily armed extremist threatened the crowd and murdered 69 people on Utøya Island, Norway. At 3 months after the terrorist attack, the 490 survivors who were at least 13 years of age were sent postal invitations to participate in the present study and were subsequently contacted by telephone. Of these, 165 survivors could not be reached by telephone or declined to participate. As a result, 325 (66.3%) survivors were interviewed face to face, most of them in their homes (Dyb *et al.* 2014; Hafstad *et al.* 2014). There were no significant differences between the gender, age, hospitalization, or region of residence of participants and non-participants (Stene & Dyb, 2016). During the massacre, the young victims' parents were bystanders, following events through the media or through telephone contact with their children without being able to intervene. The media coverage was extensive during and immediately after the attack, and a large proportion of the youth called their parents to say a last goodbye or to ask for help and advice on how to escape the perpetrator. Although none of the parents was in danger, they were exposed to significant trauma by experiencing a threat to their children's lives. Parents of all survivors aged 33 years and below were invited to participate in face-to-face interviews or in a postal survey. At wave 1, 453 parents took part, while 426 took part at wave 2, 1 year later. The study was based on written consent and was approved by the Regional Committee for Medical and Health Research Ethics in Norway.

### Measures

#### PTSD

Post-traumatic stress reactions over the past month were measured in both samples using the UCLA PTSD Reaction Index for DSM-IV (PTSD-RI) (Pynoos *et al.* 1998; Steinberg *et al.* 2004, 2013). The 17 PTSD items are endorsed on a five-point scale, ranging from 0 (never) to 4 (most of the time). In addition to these items, 11 questions were added to assess three new DSM-5 symptom criteria, as proposed by the original authors of the scale (Pynoos & Steinberg, 2013). As these criteria have several alternative formulations, they are assessed with three (D2), two (D3) and four (D4) items, respectively, and the item with the highest score is applied as an indicator for the specific symptom criteria. When combined, these items add up to 20 criteria, corresponding to the DSM-5. Due to the characteristics of the event, all survivors and parents were considered to meet criterion A. To determine diagnostic caseness, we used the conservative approach suggested by Steinberg *et al.* (2013), requiring a score of 3 (much of the time) or 4 (most of the time) for a symptom to be considered present, and then followed the DSM-5 algorithm of at least two B, one C, two D and two E criteria for a tentative diagnosis to be met.

Proposed ICD-11 diagnostic guidelines were approximated by operationalizing the requirements of (B) re-experiencing the TE in the form of either vivid intrusive memories, flashbacks, or nightmares, (C) avoidance of thoughts and memories of the TE or of activities or situations reminiscent of the TE, and (D) excessive hypervigilance or enhanced startle reactions (see Table 1 for an overview of all symptoms and their mapping to each of the diagnostic systems).We tested two different diagnostic algorithms reflecting different suggestions on how to operationalize the ICD-11 re-experiencing criterion; model 1 included intrusive memories in addition to flashbacks and nightmares, and model 2 comprised only flashbacks and nightmares. To facilitate comparison with the DSM-5 diagnosis, we used the same conservative requirement of a score of 3 or 4 for a symptom to be considered present, and then followed the ICD-11 proposal that at least one B, one C and one D criterion should be met for a tentative diagnosis.

**Table 1.** PTSD criteria and symptom mapping for different diagnostic conceptualizations

Symptom criteria	DSM-5	ICD-11 model 1	ICD-11 model 2
Intrusive memories	Re-experiencing	Re-experiencing^a^	
Nightmares	Re-experiencing	Re-experiencing	Re-experiencing
Flashbacks	Re-experiencing	Re-experiencing	Re-experiencing
Psychological reactivity to reminders	Re-experiencing		
Physiological reactivity to reminders	Re-experiencing		
Avoidance of thoughts/feelings	Avoidance	Avoidance	Avoidance
Avoidance of situations/people	Avoidance	Avoidance	Avoidance
Trauma-related amnesia	Cognition and mood		
Negative beliefs	Cognition and mood		
Blame	Cognition and mood		
Pervasive negative emotional state	Cognition and mood		
Loss of interest	Cognition and mood		
Feeling detached	Cognition and mood		
Difficulty experiencing positive emotions	Cognition and mood		
Irritability or anger outbursts	Hyperarousal		
Reckless behaviour	Hyperarousal		
Hypervigilance	Hyperarousal	Hyperarousal	Hyperarousal
Exaggerated startle response	Hyperarousal	Hyperarousal	Hyperarousal
Difficulty concentrating	Hyperarousal		
Sleep difficulties	Hyperarousal		

PTSD, Post-traumatic stress disorder; DSM-5, Diagnostic and Statistical Manual for Mental Disorders, fifth edition; ICD-11, International Classification of Diseases-11.

a For a re-experiencing symptom to be considered as being present, a score of 4 (most of the time) is required. For all other symptoms, a score of 3 or 4 is required. Diagnostic algorithms: DSM-5: ⩾2 re-experiencing, ⩾1 avoidance, ⩾2 cognition and mood, and ⩾2 hyperarousal; ICD-11 versions 1 and 2: ⩾1 re-experiencing, ⩾1 avoidance, and ⩾1 hyperarousal.

#### Symptoms of anxiety and depression

Levels of depression and anxiety over the past 2 weeks were measured based on the eight-item version of the Hopkins Symptom Checklist (HSCL-8), rated on a scale from 1 (not bothered) to 4 (bothered a great deal), and applied as a mean score. Shorter versions of the SCL have shown good psychometric properties (Tambs & Moum, 1993; Strand *et al.* 2003). In the present study, Cronbach's *α* for the survivor sample was 0.85 for the total mean scale at time 1 and 0.89 at time 2, and 0.91 and 0.92 for the parent sample at time 1 and time 2, respectively.

#### Functional impairment

Following the assessment of current symptoms, functional impairment at time 1 was measured based on three items that were designed for this study: (1) Do you find it difficult to get things done?; (2) Do you find it difficult to get along with or be with family and friends?; and (3) Are you much less interested in, or are unable to do things you used to do before July 22nd?. Items were rated on a five-point scale, from 0 (not at all) to 4 (most of the time) and a mean score was applied, with higher scores indicating a greater functional impairment. Cronbach's *α* was 0.74 for the survivor sample and 0.80 for the parent sample. At time 2, functional impairment was determined by assessing the degree to which participants felt that their level of functioning had returned to normal within the following five settings/domains: (1) school, (2) being with friends, (3) being with family, (4) extracurricular activities, and (5) household duties. Items were scored from 1 (totally back to normal) to 5 (not at all back to normal), and a mean score was calculated. Cronbach's *α* for the scale was 0.81 for the survivor sample and 0.87 for the parent sample.

#### Life satisfaction

Life satisfaction was measured by Cantril's self-anchoring scale (Cantril, 1965). This scale is constructed as a ladder that ranges from 1 to 10, where 1 reflects ‘the worst imaginable life’ and 10 reflects ‘the best imaginable life’. Life satisfaction was used as an indication of the overall influence of symptoms on daily life and functioning.

### Statistical analyses

We estimated agreement between the ICD-11 and DSM-5 diagnoses using Cohen's *κ*, and we used exact McNemar tests to check for significant differences in prevalence rates across the diagnostic algorithms. To test the predictive validity of each scoring algorithm, we examined the relationship between the diagnosis and symptoms of anxiety and depression (HSCL-8), functional impairment and life satisfaction. The analysis was performed in two steps. We first conducted a set of independent-sample *t* tests to assess whether the diagnostic cases and non-cases within each diagnostic algorithm differed in mean levels of HSCL-8, functional impairment and quality of life, and then whether the different algorithms produced smaller or larger differences between the measures of interest. In this way, we assessed whether the diagnostic algorithms differed in their ability to distinguish between individuals with a higher symptom load, higher functional impairment and lower life satisfaction. We then used a bootstrap procedure to produce 95% bias-corrected and accelerated confidence intervals for each difference calculation, with results considered to be significant if 0 was outside the interval. All calculations were performed for both samples and at both measurement points. To test the predictive power of the diagnostic algorithms over time, we repeated the same bootstrap procedure to determine whether ICD-11 and DSM-5 differed in their ability to distinguish between individuals with a higher symptom load, higher functional impairment and lower life satisfaction 1 year later. The number of missing values was generally very low. Therefore, for all scale scores, missing values were replaced by the mean of the individual's scores on the scale. All analyses were performed using SPSS version 22.0 and R version 3.1.2 (The R Foundation for Statistical Computing, Austria) with the R package ‘boot’ for bootstrapping.

## Results

### Do ICD-11 and DSM-5 give the same PTSD prevalence?

Overall, use of the DSM-5 criteria resulted in the highest PTSD prevalence (Table 2). In the parent sample, ICD-11-based PTSD prevalence was significantly lower than the DSM-5 prevalence at both time points, regardless of whether intrusive memories were included or not. For survivors, the picture looked somewhat different. At wave 1 no significant differences were found between the levels of PTSD and at wave 2, the rate of DSM-5-based PTSD was significantly higher only when intrusive memories were excluded from the ICD-11 model (model 2: 5.6% *v.* 8.4%, *p* = 0.008).

**Table 2.** Prevalence of PTSD using different DSM-5 and ICD-11 algorithms in both samples at wave 1 and wave 2

	Sample 1: survivors		Sample 2: parents	
Diagnoses	Wave 1 (4–5 months) (*n* = 325)	Wave 2 (14–15 months) (*n* = 285)	Wave 1 (4–5 months) (*n* = 451)	Wave 2 (14–15 months) (*n* = 426)
DSM-5	38 (11.7)	24 (8.4)	29 (6.4)	28 (6.6)
ICD-11 model 1 (including intrusive memories)	35 (10.8)	18 (6.3)	17 (3.2)	16 (3.8)
ICD-11 model 2 (excluding intrusive memories)	32 (9.8)	16 (5.6)	16 (3.0)	14 (3.3)

Data are given as number of participants (percentage).

PTSD, Post-traumatic stress disorder; DSM-5, Diagnostic and Statistical Manual for Mental Disorders, fifth edition; ICD-11, International Classification of Diseases-11.

### Do ICD-11 and DSM-5 identify the same individuals?

Overall, the overlap between those diagnosed with the ICD-11 and the DSM-5 was modest. In the survivor sample at wave 1, 26 individuals (8.0% of the total sample) met the criteria for both DSM-5 and ICD-11, while 19 (5.8%) met the criteria for one diagnosis only; 10 met only the DSM-5 criteria and nine only the ICD-11 criteria (model 1). Cohen's *κ* between the diagnoses was 0.70, suggesting a ‘fair to good’ overlap. Values were very similar for the ICD-11 model 2 which does not include intrusive memories. At wave 2, 16 individuals (5.6% of the total sample) met the criteria for both diagnoses, while 10 (3.5%) met the criteria for one diagnosis only. Only two of those who met the ICD-11 (model 1) diagnosis did not meet the DSM-5 diagnosis, and Cohen's *κ* between the systems was 0.74.

The overlap among parents was even smaller. At wave 1, 14 (3.1% of the total sample) met the diagnostic criteria for both ICD-11 and DSM-5, while several more met the criteria for one of the diagnoses only (*n* = 18, 4.0% of the total sample). More than half (*n* = 15, 51.7%) of those who met the DSM-5 criteria did not meet the ICD-11 criteria, while three (17.0%) of those who met the proposed ICD-11 criteria did not meet the DSM-5 criteria. Values were similar for wave 2; 28 individuals (6.6% of the total sample) met the criteria for both diagnoses, while 16 met the criteria for only one. Cohen's *κ* was 0.62. Again, the DSM-5 was more inclusive: only two of those who met the ICD-11 criteria did not meet the DSM-5, while 14 of those who met the DSM-5 criteria did not meet the ICD-11 criteria.

### Are ICD-11 and DSM-5 equally good at identifying individuals with significant distress and functional impairment?

To investigate the predictive power of the two diagnostic algorithms, we examined the degree to which cases and non-cases, according to the ICD-11 and DSM-5, differed in level of anxiety and depression, functional impairment and life satisfaction. Overall, there were few significant differences between the systems' abilities to identify individuals with higher levels of distress and impairment. Among survivors, PTSD cases identified by DSM-5 algorithms had higher levels of functional impairment at wave 1 compared with those identified by ICD-11 (Table 3). It did not matter whether intrusive memories were included in the re-experiencing criterion or not (i.e. whether we used ICD-11 model 1 or 2).

**Table 3.** Concurrent predictive power of the diagnostic algorithms in sample 1 survivors at wave 1 and wave 2

	Wave 1				Wave 2			
	ICD-11 model 1 *v.* DSM-5		ICD-11 model 2 *v.* DSM-5		ICD-11 model 1 *v.* DSM-5		ICD-11 model 2 *v.* DSM-5	
	Diff (s.e.)	95% CI	Diff (s.e.)	95% CI	Diff (s.e.)	95% CI	Diff (s.e.)	95% CI
Anxiety/depression	−0.03 (0.07)	−0.19 to 0.09	0.16 (0.12)	−0.07 to 0.40	0.16 (0.11)	−0.07 to 0.40	0.12 (0.10)	−0.03 to 0.38
Impairment	−0.27 (0.00)	−0.57 to −0.03*	0.05 (0.11)	−0.15 to 0.28	0.05 (0.10)	−0.14 to 0.28	−0.07 (0.13)	−0.39 to 0.14
Life satisfaction	0.02 (0.21)	−0.47 to 0.39	−0.32 (0.42)	−1.24 to 0.42	−0.32 (0.41)	−1.23 to 0.41	−0.26 (0.46)	−1.22 to 0.59

ICD-11, International Classification of Diseases-11 DSM-5, Diagnostic and Statistical Manual for Mental Disorders, fifth edition; Diff, difference scores between ICD cases and DSM cases on mean levels of anxiety/depression, impairment and life satisfaction; s.e., standard error; CI, confidence interval.

* Significant difference between ICD-11 and DSM-5 in detecting discrepant outcome variable scores in cases and non-cases.

In parents at wave 1, cases meeting the DSM-5 diagnostic criteria reported lower levels of life satisfaction than cases identified by either of the ICD-11 algorithms (Table 4). At the same time, meeting the ICD-11 criteria (model 2 only) was associated with higher levels of anxiety and depression.

**Table 4.** Concurrent predictive power of the diagnostic algorithms in sample 2 parents at wave 1 and wave 2

	Wave 1				Wave 2			
	ICD-11 model 1 *v.* DSM-5		ICD-11 model 2 *v.* DSM-5		ICD-11 model 1 *v.* DSM-5		ICD-11 model 2 *v.* DSM-5	
	Diff (s.e.)	95% CI	Diff (s.e.)	95% CI	Diff (s.e.)	95% CI	Diff (s.e.)	95% CI
Anxiety/depression	0.04 (0.11)	−0.19 to 0.27	0.03 (0.12)	0.21 to 0.28*	0.13 (0.12)	−0.09 to 0.40	0.09 (0.14)	−0.16 to 0.37
Impairment	−0.06 (0.16)	−0.36 to 0.42	−0.07 (0.17)	−0.39 to 0.29	−0.08 (0.17)	−0.40 to 0.27	−0.30 (0.18)	−0.68 to 0.05
Life satisfaction	−0.34 (0.37)	−1.10 to −0.40*	−0.24 (0.42)	−1.06 to 0.60	−0.28 (0.47)	−1.22 to 0.66	−0.08 (0.51)	−1.07 to 0.93

ICD-11, International Classification of Diseases-11 DSM-5, Diagnostic and Statistical Manual for Mental Disorders, fifth edition; Diff, difference scores between ICD cases and DSM cases on mean levels of anxiety/depression, impairment and life satisfaction; s.e., standard error; CI, confidence interval.

* Significant difference between ICD-11 and DSM-5 in detecting discrepant outcome variable scores in cases and non-cases.

Finally, we investigated the predictive power of the diagnostic algorithms over time, that is, whether being diagnosed with PTSD by either the DSM-5 or the ICD-11 criteria after 5 months (wave 1) could predict overall levels of functioning 1 year later. We examined the degree to which diagnostic status according to the proposed ICD-11 and DSM-5 at wave 1 predicted differences in level of anxiety and depression, functional impairment and life satisfaction at wave 2. Again, there were no significant differences between ICD-11 (either of the models) and DSM-5 in their ability to detect impairment over time, that is, they did not differ significantly in predictive power over time (for results, see online Supplementary Tables S1 and S2). All in all, the findings from the study do not suggest that one diagnostic system was superior to the other in identifying clinically significant cases.

## Discussion

How PTSD can best be conceptualized has been heavily debated over the past three decades. The fact that the two major diagnostic systems have come to quite divergent solutions reflects the challenges we still face in understanding the phenomenon of PTSD. This study evaluated the implications of the DSM-5 PTSD criteria and two alternative operationalizations of the proposed ICD-11 criteria for PTSD. We found that, while diagnostic prevalence was comparable for the ICD-11 and DSM-5 diagnoses among survivors, the ICD-11-based prevalence was significantly lower than the DSM-5-based prevalence in parents. The two systems diagnosed somewhat different individuals, although the overlap was much greater for survivors than for parents. Findings regarding predictive validity revealed few significant differences between ICD-11 and DSM-5.

### Prevalence and concordance between PTSD in DSM-5 and ICD-11

So far a handful of studies have compared PTSD prevalence using the DSM-5 and the proposed ICD-11 criteria (O'Donnell *et al.* 2014; Stein *et al.* 2014). In line with the main findings from the survivor sample, Stein *et al.* (2014) found no significant difference in prevalence between the two diagnostic systems. O'Donnell *et al.* (2014) found that applying the DSM-5 criteria produced a significantly higher last-month prevalence than the ICD criteria, which is in accordance with our findings from the parent sample. The same was true for the Hyland *et al.*'s (2016) study of child sexual abuse survivors, as well as Hansen and collaborators' study of seven different trauma samples (Hansen *et al.* 2015).

Although we noticed that the ICD model including intrusive memories identified more individuals with PTSD than the model without this criterion, the diagnostic prevalence did not differ significantly between the two ICD-11 models. However, the finding at wave 2 showing that the DSM-5 prevalence rate differed significantly from the rate identified by the ICD-11 model without intrusive memories suggests that this criterion may play an important role in identifying PTSD. This finding relates to what O'Donnell *et al.* (2014) found in their sample of injured males and Hyland *et al.* (2016) found in their sample of female survivors of child sexual abuse. It seems that adding intrusive memories to the ICD-11 diagnosis might make the diagnostic systems more comparable.

In our parent sample, the ICD-11 produced a significantly lower PTSD prevalence than the DSM-5 algorithm, at both waves. At wave 1, the prevalence according to ICD-11 was about half of the prevalence according to DSM-5. The differences in the youth sample were not significant, although the number of individuals identified with PTSD using ICD-11 was lower than with DSM-5 at both waves. Moreover, the overlap between the systems was disturbingly low in both samples, but especially in the parent sample. The experiences related to the event were, of course, different for parents than for their children. The survivors' lives were directly threatened at the island but parents had more diverse experiences, possible resulting in more diverse symptoms, not necessarily the suggested (ICD-11) core symptoms of PTSD. Rather, it could be that they experience other symptoms of emotional distress, such as depressive symptoms, which would probably be covered by the DSM-5 PTSD criteria, or prolonged grief disorder – a related disorder proposed for ICD-11 that specifically covers bereavement-related symptoms (Maercker *et al.* 2013*a*).

The ICD is the official clinical diagnostic system in use worldwide, apart from the USA, but most research is based on the DSM. Large differences in these diagnostic systems may reduce the relevance of research for clinical practice if the diagnostic approaches do not identify the same group of individuals. Thus, one major question is whether the discrepancy between the diagnoses has increased or decreased after the revision of the symptom criteria. Studies comparing changes between ICD-10 and the proposed ICD-11 have shown reduced prevalence of PTSD, indicating that the new criteria are either more specific or less sensitive or both. The lack of convergence between PTSD diagnoses in the DSM and the ICD has been an issue within the trauma field. Whereas previous evidence suggests that prevalence estimates of DSM-IV and ICD-10 PTSD are quite similar (Morina *et al.* 2014), Stein *et al.* (2014) found that the difference in prevalence between the ICD-11 and DSM-5 was actually smaller than the difference between the ICD-10 and the DSM-IV in the same sample, suggesting a harmonizing of the diagnoses. However, the systems still identify somewhat distinct sets of individuals, which was also an issue in the current study.

### Prediction of anxiety and depression, functional impairment and life satisfaction

One of the main aims of this study was to evaluate the predictive validity of the proposed ICD-11 diagnosis and the DSM-5 diagnosis. In accordance with the findings from O'Donnell *et al.* (2014), who studied an Australian adult traumatic injury sample, we found that there were small and inconsistent differences in the predictive validity of the DSM-5 *v.* the ICD-11 diagnosis. This may indicate that the systems are equally reliable for identifying individuals in need of mental health interventions.

Harmonizing the diagnostic systems may benefit patients across the world, and extensive efforts have been made to reach that goal. Following the recent revisions, a major question is whether the discrepancy between the diagnostic systems has increased or decreased. Studies comparing changes between ICD-10 and the proposed ICD-11 have shown reduced prevalence of PTSD, indicating that the new criteria are either more specific or less sensitive or both. Previous evidence has suggested that the PTSD prevalence estimates of DSM-IV and ICD-10 were quite similar (Morina *et al.* 2014), and Stein *et al.* (2014) found even greater similarity between the ICD-11 and DSM-5 in the same sample. However, as shown in this study, distinct differences between the systems also seem to result in disturbingly low overlap in the individuals being diagnosed across systems. For individuals suffering from post-traumatic stress, diagnosis may be a gateway to health services, compensation and treatment choices. It is likely that the differences in the DSM and the ICD systems will have practical implications for many individuals in the years to come.

### Strengths and limitations

This study had several strengths including repeated assessments in two samples at two time points, allowing for multiple comparisons of the diagnostic algorithms, as well as the high response rate and low levels of missing data. We also had some limitations that should be addressed in future research. Although the UCLA PTSD index is a validated instrument, it has not been validated in Norwegian. Moreover, we did not include the distress and impairment symptoms or the physiological exclusion criteria for PTSD. Therefore, we could not estimate the true diagnostic prevalence, but rather a probable diagnostic status. As well, the proposed ICD-11 diagnostic guidelines are not yet written as research criteria and we needed to approximate them by using criteria specified in the DSM. This, along with the fact that we used a clinician-administrated fully structured interview, rather than a clinical interview, may have made an impact on the estimation of the ICD-11 PTSD prevalence. The way functional impairment was assessed in the first wave was less than optimal, as some of the items may have been conflated with some of the dysphoric PTSD symptoms. We acknowledge that we might have better captured impairment by using a more comprehensive measure at this time point. This was improved at the second wave, where we included a more precise and comprehensive measure. Another weakness relates to the way we conceptualized some of the ICD-11 criteria in this study. The re-experiencing symptom as defined in the DSM-5 differs somewhat from the one proposed for the ICD-11. It is therefore questionable whether we captured the re-experiencing or intrusion symptom as described in the ICD-11 definition, i.e. ‘re-experiencing the traumatic event in the present in the form of vivid intrusive memories, accompanied by fear, horror, flashbacks and nightmares’. Adding a re-experiencing symptom assessing this particular feature of re-experiencing to a greater extent may have added additional validity to the ICD-11 findings. This limitation relates primarily to model 1; model 2, which includes intrusive memories, is very similar to the re-experiencing criteria in DSM-5. It is also possible that the agreement between the diagnoses was optimalized (and higher than it would be in clinical practice) because we used standardized cut-off values for single items, and because the questions on which we based the ICD-11 diagnosis were not derived from a clinical interview, but from the same self-report form on which the DSM-5 diagnosis was based. Finally, it must be noted that the event was extraordinary in several ways, not least in its brutality, but also in that the surviving youth live in a country with a well-functioning health care system. As such, our findings may not be generalizable to a wide range of trauma populations. That said, the particular features of this event and the sample add to the literature by showing how the diagnostic systems work under very particular circumstances.

## Conclusion

The diagnostic systems performed somewhat differently in assessing last-month prevalence rates, but were relatively similar in how well they predicted level of functioning and life satisfaction. The overlap was relatively low between those diagnosed with PTSD by the ICD-11 and DSM-5 criteria and a substantial proportion met only one set of criteria. This represents a challenge for research and for clinical work for two reasons: (1) research based on DSM criteria may become less useful for clinical work based on ICD diagnostics around the world and (2) clinical practice using ICD may select individuals for trauma-specific treatments developed and proven effective by DSM-based research.

Working to understand the true nature of PTSD is crucial for better use of research resources and for optimally efficient and fair patient treatment. Future studies need to focus on functional measures and treatment outcomes to determine which of the diagnostic systems is more valid. One way to do this would be to include both sets of diagnostic criteria in clinical studies and monitor how diagnostic status according to both is associated with outcome of trauma-specific treatment.

## Supplementary material

For supplementary material accompanying this paper visit http://dx.doi.org/10.1017/S0033291716002968. click here to view supplementary material
