# Supplementary material for: PTSD or not PTSD? Comparing the proposed ICD-11 and the DSM-5 PTSD criteria among young survivors of the 2011 Norway attacks and their parents
Source: Psychol Med. 2017 Jan 12;47(7):1283–91. doi: 10.1017/S0033291716002968 (PMC5426334; doi:10.1017/S0033291716002968)
Supplement: Supplementary file 1 [file S0033291716002968sup001.zip › S0033291716002968sup001.doc]

***Supplementary Table S1.*** Predictive power of the diagnostic algorithms over time, sample 1 survivors

|  | T1-T2: Sample 1 survivors | | | | | | | |
| --- | --- | --- | --- | --- | --- | --- | --- | --- |
|  | ICD-11 T1 Mod 1* | | DSM-5 T1 | | Difference in detecting impaired functioning | | | |
|  | Case | Non-case | Case | Non-case | ICD-11 Mod 1 v DSM-5 | | ICD-11 Mod 2 v DSM-5 | |
|  | M (sd) | M (sd) | M (sd) | M (sd) | Diff (SE) | CI (95%) | M (sd) | M (sd) |
| Anxiety/depression Wave 2 | 2.26 (0.15) | 1.76 (0.04) | 2.30 (0.77) | 1.75 (0.62) | -0.042 (0.10) | -0.24, 0.16 | -0.01 (0.11) | (-0.23,0.21) |
| Impairment Wave 2 | 2.45 (0.88) | 1.90 (0.73) | 2.48 (0.93) | 1.88 (0.72) | -0.032 (0.13) | -0.29, 0.22 | -0.01 (0.14) | (-0.29,0.25) |
| Life satisfaction Wave 2 | 6.83 (2.26) | 7.29 (1.92) | 6.85 (2.13) | 7.29 (1.93) | -0.01 (0.27) | -0.51, 0.53 | -0.10 (0.29) | (-0.69,0.45) |

*Results were similar for Model 2. There were no significant differences between DSM-5 and ICD-11 in predictive power over time.
