# Supplementary material for: PTSD or not PTSD? Comparing the proposed ICD-11 and the DSM-5 PTSD criteria among young survivors of the 2011 Norway attacks and their parents
Source: Psychol Med. 2017 Jan 12;47(7):1283–91. doi: 10.1017/S0033291716002968 (PMC5426334; doi:10.1017/S0033291716002968)
Supplement: Supplementary file 1 [file S0033291716002968sup001.zip › S0033291716002968sup002.doc]

***Supplementary Table S2.*** *Predictive power of the diagnostic algorithms over time, sample 2 parents*

|  | T1-T2: Sample 2 parents | | | | | | | |
| --- | --- | --- | --- | --- | --- | --- | --- | --- |
|  | ICD-11 T1 Mod 1* | | DSM-5 T1 | | Difference in detecting impaired functioning | | | |
|  | Case | Non-case | Case | Non-case | ICD-11 Mod 1 v DSM-5 | | ICD-11 Mod 2 v DSM-5 | |
|  | M (sd) | M (sd) | M (sd) | M (sd) | Diff (SE) | CI (95%) | M (sd) | M (sd) |
| Anxiety/depression Wave 2 | 2.49 (0.75) | 1.56 (0.60) | 2.41 (0.66) | 1.51 (0.55) | 0.09 (0.14) | (-0.17,0.37) | 0.12 (0.19) | (-0.24,0.49) |
| Impairment Wave 2 | 2.85 (0.89) | 1.61 (0.74) | 2.74 (0.84) | 1.54 (0.68) | -0.30 (0.19) | (-0.68,0.04) | 0.09 (0.20) | (-0.27,0.53) |
| Life satisfaction Wave 2 | 5.31 (2.32) | 7.24 (1.20) | 6.05 (2.04) | 7.30 (1.90) | -0.86 (0.51) | (-1.07,0.95) | 0.09 (0.42) | (-0.71.0.98) |

* Findings were similar for Model 2. There were no significant differences between ICD-11 and DSM-5 in predictive power over time.
